# Crystal structure, Hirshfeld surface analysis and electrostatic potential study of naturally occurring cassane-type diterpenoid Pulcherrimin C monohydrate at 100 K

**DOI:** 10.1107/S2056989018017498

**Published:** 2019-01-04

**Authors:** Rajesh Kumar, K. Osahon Ogbeide, Mujeeb-Ur-Rehman, Bodunde Owolabi, Abiodun Falodun, M. Iqbal Choudhary, Sammer Yousuf

**Affiliations:** a H. E. J. Research Institute of Chemistry, International Center for Chemical and Biological Sciences, University of Karachi, Karachi-75270, Pakistan; b Department of Chemistry, Faculty of Physical Sciences, University of Benin, Benin City, Nigeria; c Department of Chemistry, School of Sciences, The Federal University of Technology, Akure, Nigeria; d Department of Pharmaceutical Chemistry, Faculty of Pharmacy, University of Benin, Benin City, Nigeria

**Keywords:** *Caesalpinia pulcherrima*, cassane-type diterpenoids, pulcherrimin C, Hirshfeld surface analysis, electrostatic potential, crystal structure

## Abstract

The title cassane-type diterpenoid known as pulcherrimin C, C_34_H_36_O_8_·H_2_O, systematic name 5,6-bis­(benzo­yloxy)-4a-hy­droxy-4,7,11b-trimethyl-1,2,3,4,4a,5,6,6a,7,11,11a,11b-dodeca­hydro­phenanthro[3,2-*b*]furan-4-carb­oxy­lic acid monohydrate, was isolated as a monohydrate from the medicinally important plant *Caesalpinia pulcherrima*, found in the tropical regions of south and south-east Asia. The mol­ecule is composed of three *trans*-fused six-membered rings having chair, chair and half-chair conformations, and a five-membered planar furan ring. In the crystal, O—H⋯O hydrogen bonds link mol­ecules into chains parallel to the *b* axis. Weak C—H⋯π inter­actions are also observed. Hirshfeld surface analysis indicates that the contribution of O⋯H inter­actions towards the total generated Hirshfeld surface is 21.5%.

## Abstract 2

Single crystal X-ray diffraction analysis and Hirshfeld surface analysis of the title compound were carried out to analyse qu­anti­tatively the inter­molecular inter­actions involved in the crystal packing. The electrostatic potential surface was generated over the Hirshfeld surface to visualize the potential active sites.

## Chemical context   

*Caesalpinia pulcherrima* (L) Swartz is an enduring shrub or small tree of the cassane family found in tropical regions of south and south-east Asia. It has been used ornamentally for a long time and is commonly known as Paradise flowers, Pride of Barbados and Peacock flower (Quisumbing, 1951 ▸). In addition, its parts have also been utilized as a traditional medicine in Thailand. The flowers and leaves are believed to be a cure for fever (Lotschert *et al.*, 1983 ▸), and people in the northern regions of Thailand use its roots to treat tuberculous symptoms (Wutthithammaweach *et al.*, 1997 ▸). Furthermore, it has also been proved that its crude DCM extract exhibits relatively strong anti-tubercular activity (Promsawan *et al.*, 2003 ▸). A methanol extract of *C. pulcherrima* has been reported to have strong anti­bacterial activity (Parekh *et al.*, 2006 ▸). The plant is also used to treat cardiovascular disorders, inflammation, muscular and sore pain, earache, and is known for its anti­pyretic, vermifugal and anti­malarial activities (Patel *et al.*, 2010 ▸; Roach *et al.*, 2003 ▸). The present investigation deals with the isolation, single-crystal X-ray diffraction study, Hirshfeld surface analysis and electrostatic potential studies of the naturally occurring title compound, which was isolated as a monohydrate.
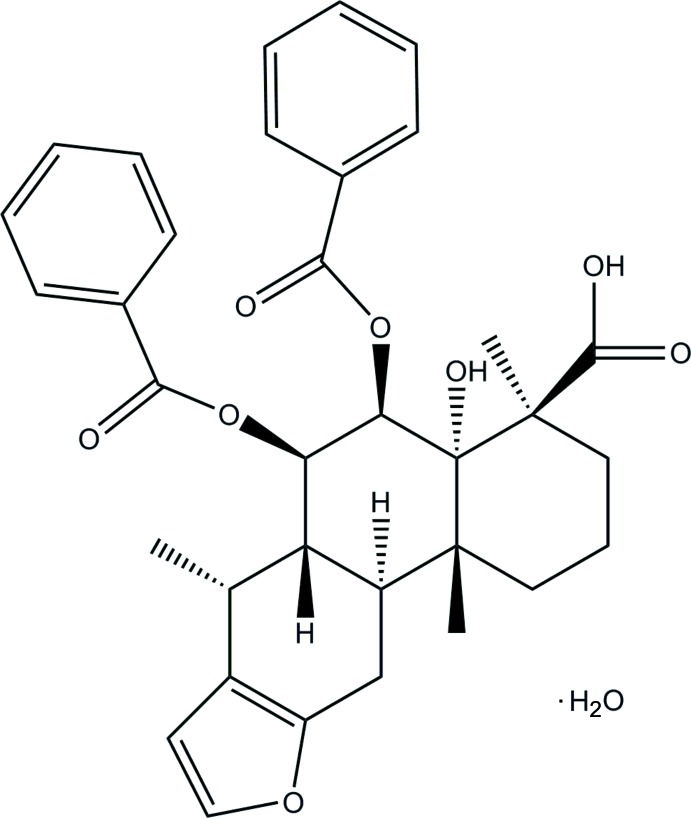


## Structural commentary   

**Figure 1:**
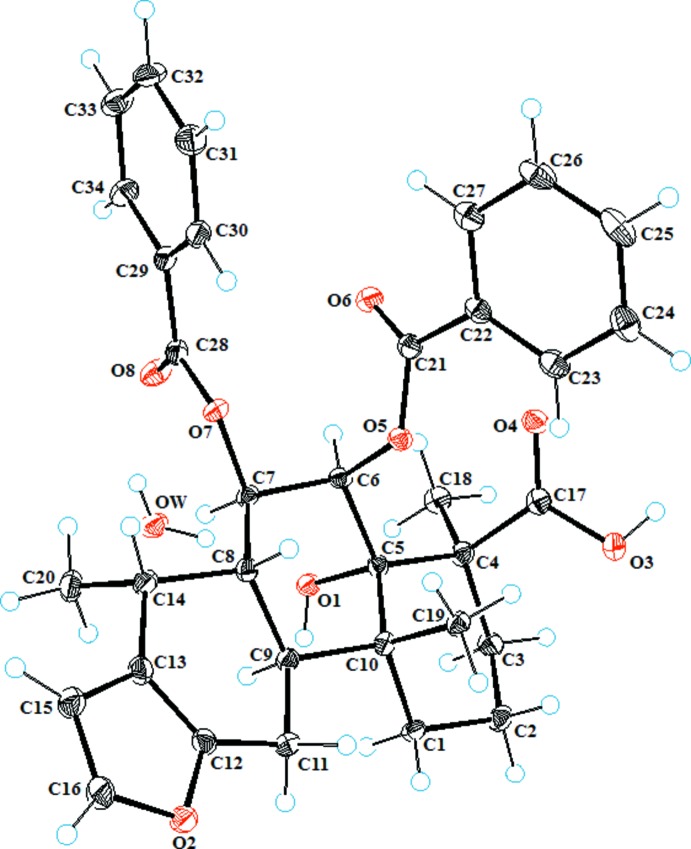
The mol­ecular structure of the title compound with displacement ellipsoids drawn at the 50% probability level.

The mol­ecule of the title compound (Fig. 1 ▸) consists of three trans-fused rings, *A* (C1–C5/C10), *B* (C5–C10) and *C* (C8/C9/C11–C14) having chair, chair and half-chair confirmations; the puckering parameters are *Q* = 0.554 (3) Å, θ = 6.9 (3)°, φ = 6(3)° for *A*; *Q* = 0.591 (3) Å, θ = 0.0 (3)°, φ = 318 (12)° for *B*; *Q* = 0.446 (3) Å, θ = 48.0 (4)°, φ = 12.4 (5)° for *C*. The adjacent cinnamoyl groups attached to atoms C6 and C7 are *cis* to each other, and the dihedral angle formed by their phenyl rings is 28.13 (10)°. The planar furan ring (O2/C12/C13/C15/C16) forms dihedral angles of 88.58 (8)° and 69.34 (10)°, respectively, with the C22–C27 and C29–C34 phenyl rings. The absolute configurations of the stereogenic centers at atoms C4, C5, C6, C7, C8, C9, C10 and C14 are established as *S*, *S*, *R*, *R*, *R*, *S*, *R* and *R* on the basis of the reported literature (Patil *et al.*, 1997 ▸).

The intra­molecular C19—H19*B*⋯O3 hydrogen bond (Table 1 ▸) forms a ring with an *S*(7) graph-set motif.

## Superamolecular features and Hirshfeld surface analysis   

**Figure 2:**
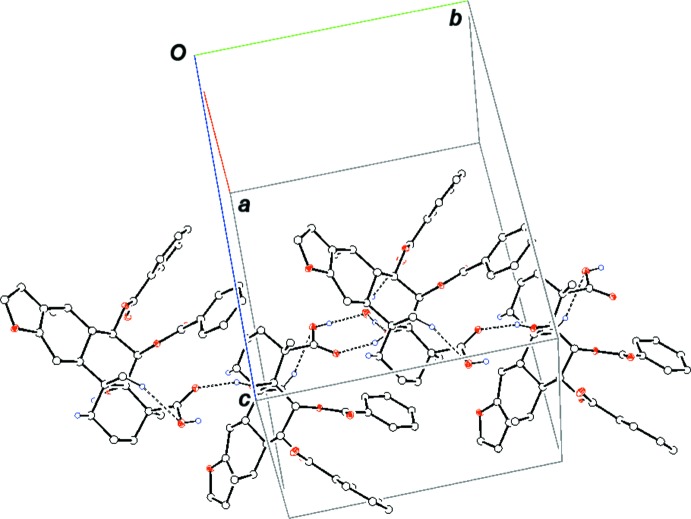
Partial packing diagram of the title compound showing the formation of a chain parallel to the *b* axis by O—H⋯O hydrogen bonds (dotted lines). Intra­molecular C—H⋯O hydrogen bonds (dotted lines) are also shown. Hydrogen atoms not involved in hydrogen bonding are omitted.

Inter- and intra­molecular inter­actions exert a significant influence on the geometry and properties of crystalline materials (Ferenczy *et al.*, 2001 ▸; Putz *et al.*, 2016 ▸). Analysis of the hydrogen bonding shows the presence of both conventional and non-conventional types of hydrogen-bonded contacts in the crystal structure of the title compound (Fig. 2 ▸, Table 1 ▸). The oxygen atom of the water mol­ecule acts as acceptor for the hydroxyl hydrogen atom of neighboring mol­ecule via O3—H3⋯O1*W* inter­actions, while the two hydrogens atoms inter­act with the hydroxyl group at atom C5 and the carbonyl functionality of neighbouring mol­ecules via O1*W*—H*WA*⋯O1 and O1*W*—H*WB*⋯O8 hydrogen bonds, forming an 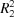
(10) ring. These inter­actions, along with the O1—H1⋯O4 hydrogen bond, link the mol­ecules into chains parallel to the *b* axis. Relatively weak C—H⋯π inter­actions (Table 1 ▸) are also observed.

**Figure 3:**
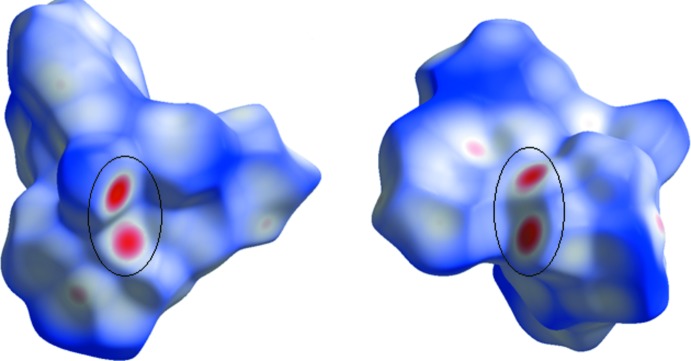
Hirshfeld surface mapped over *d*_norm_ generated for the title compound.

**Figure 4:**
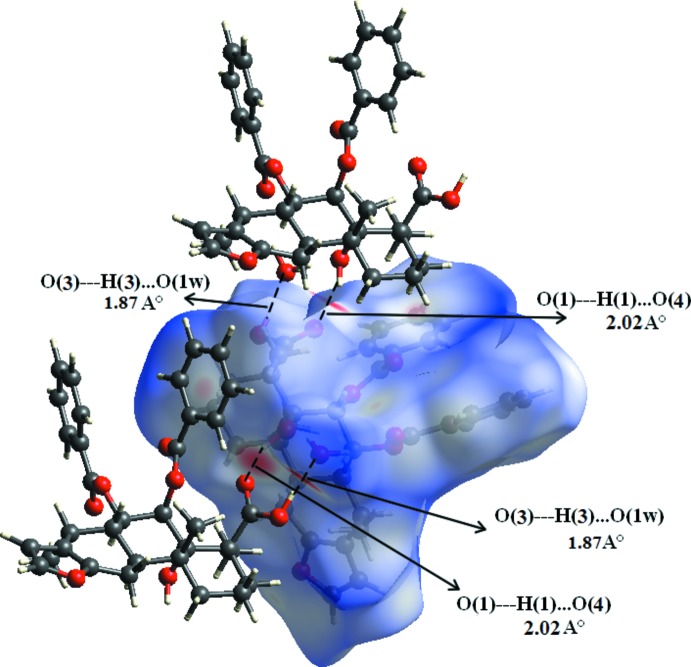
Hirshfeld surface mapped over *d*_norm_ for the title compound with neighbouring mol­ecules linked via O—H⋯O hydrogen bonds (dashed lines).

**Figure 5:**
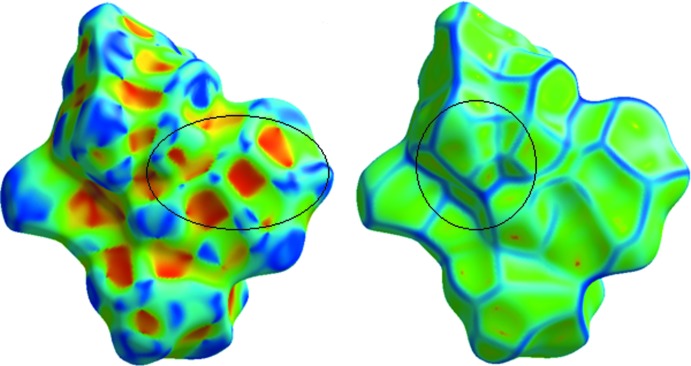
Hirshfeld surface mapped over shape-index for the title compound.

**Figure 6:**
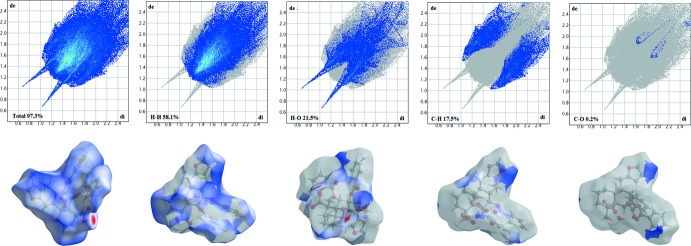
Two-dimensional fingerprint plots for the title compound together with areas of Hirshfeld surfaces involved in hydrogen bonding.

**Figure 7:**
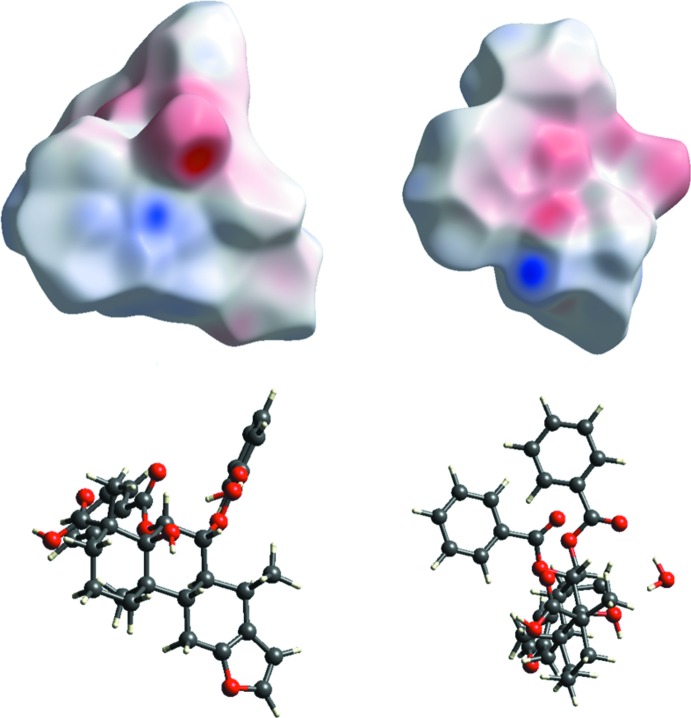
Electrostatic potential surface generated incorporated with the Hirshfeld surface for the title compound.

The three-dimensional Hirshfeld surface calculated for the title compound is depicted in Fig. 3 ▸. The red regions indicate areas of close contacts shorter than the sum of van der Waals radii, while the blue and white regions represents contacts having distances greater and equal to the sum of van der Waals radii, respectively. The O3—H3⋯O1*W* and O1—H1⋯O4 hydrogen bonds are the two inter­actions responsible for linking neighboring mol­ecules (Fig. 4 ▸). The curvedness surface (Fig. 5 ▸) shows the green (flat) and blue (curved) areas, representing low and high probabilities, respectively, of forming inter­actions with neighbouring mol­ecules. The highlighted regions shown correspond to those in Fig. 3 ▸. No obvious adjacent blue or red triangles are present, indicating the absence of π–π inter­actions. The fingerprint plots are presented in Fig. 6 ▸. H⋯H contacts are the major contributor to the Hirshfeld surface (58.1%). As a result of the presence of a water mol­ecule in the asymmetric unit, H⋯O inter­actions are observed to contribute 21.5%, with sharp spikes pointing toward the origin of the plot indicating the strength of the contacts. The contribution of C⋯H inter­actions is 17.5%, whereas C⋯O inter­actions are negligible (0.2%). The Hirshfeld surface mapped over electrostatic potential is shown in Fig. 7 ▸. The red regions indicate atoms with the potential to be hydrogen-bond acceptors (negative electrostatic potential), while blue regions indicate regions having atoms with positive electrostatic potential, *i.e.* hydrogen-bond donors.

## Database Survey   

A search of the Cambridge Structural Database (CSD version 5.39, update of August 2018; Groom *et al.*, 2016 ▸) for a common fragment composed of three *trans*-fused six-membered rings and one planar furan ring gave 13 hits, including BEQVAX {systematic name: (4a*R*,5*R*,6*R*,6a*S*,7*R*,11a*S*,11b*R*)-4a,6-dihy­droxy-4,4,7,11b-tetra­methyl-1,2,3,4,4a,5,6,6a,7,11,11a,11b-do­deca­hydro­phenanthro[3,2-*b*]furan-5-yl 3-phenyl­prop-2-en­oate; Ogbeide *et al.*, 2018 ▸), which has an α-oriented methyl substituent at C4 and axially oriented cinnamoyl and hydroxyl substituents at C6 and C7. CSLPIN10 (1,2-desacetyl-∊-caesalpin 2-*p*-bromo­benzoate; Birnbaum *et al.*, 1969 ▸) is similar to the title compound but has different substituents at various positions including C1 and C2, with α- and β-oriented methyl substituents at C4 and C10. Refcode DUTJIM {isovouacapenol C, {systematic name: (4a*R*,5*R*,6*R*,6a*S*,7*R*,11a*S*,11b*R*)-4a,6-dihy­droxy-4,4,7,11b-tetra­methyl-1,2,3,4,4a,5,6,6a,7,11,11a,11b-dodeca­hydro­phenanthro[3,2-*b*]furan-5-yl benzoate} and DUVCON {vouacapen-5α-ol, systematic name: (4a*R*,6a*S*,7*R*,11a*S*,11b*R*)-4,4,7,11b-tetra­methyl-1,2,3,4,4a,5,6,6a,7,11,11a,11b-dodeca­hydro­phenanthro[3,2-*b*]furan-4a-ol} were both also isolated from *Caesalpinia pulcherrima* (Fun *et al.*, 2010 ▸) and show hydroxyl and benzoic acid substitution at C4 and C7, respectively. Compounds EGAYIU, EGAYUG, EGAZAN and EGAZER (Jiang *et al.*, 2002 ▸), MEYREN, MEYRIR, MEYROX and MEYRUD (Jiang *et al.*, 2001 ▸) and POPNIR (Kitagawa *et al.*, 1994 ▸) all belong to the same class of compounds as the title compound, *i.e*. cassane-type diterpenoids, with different substitution patterns for the fused rings.

## Isolation and crystallization   

Fractions of the powdered stem bark of *Caesalpinia pulcherrima* were obtained according to the reported procedure (Ogbeide *et al.*, 2018 ▸). Subfraction CP124–135 (755 mg) was chromatographed on silica gel (SiO_2_, 2.5 × 70cm) and eluted isocratically with 20% ethyl­acetate in *n*-hexane to obtain a crystalline material, which was filtered and dried to give the purified title compound (226 mg) known as pulcherrimin C. Crystals suitable for X-ray analysis were obtained by slow evaporation of an ethanol solution at 296 K.

^1^H NMR (400 MHz C_3_D_6_O): 7.84 (2H, *m*), 7.79 (2H, *m*), 7.55 (2H, *m*), 7.55 (2H, *m*), 7.43 (1H, *m*), 7.33 (1H, *m*), 7.27 (1H, *d*, *J* = 1.6 Hz), 6.21 (1H, *d*, *J* = 1.6 Hz), 6.18 (1H, *d*, *J* = 3.6 Hz), 5.90 (1H, *bb*, *J* = 11.4 Hz, 3.8 Hz), 2.78 (1H, *m*), 2.66 (1H, *m*), 2.59 (1H, *m*), 2.46 (1H, *m*), 2.31 (1H, *m*), 1.50 (1H, *m*), 1.93 (1H, *m*), 1.62 (1H, *m*), 1.89 (1H, *m*), 1.79 (2H, *d*, *J* = 13.6 Hz, 4.0 Hz), 1.57 (3H, *s*), 1.41 (3H, *s*), 0.99 (3H, *d*, *J* = 6.8 Hz). IR (cm^−1^): 3527.7, 2955.9, 1718.4, 1639.9, 1456.7, 1383.4, 1283.0, 1169.0, 1109.4, 1015.4, 966.5, 799.7, 715.1.

## Refinement   

Crystal data, data collection and structure refinement details are summarized in Table 2 ▸. The water H atoms were located in a difference-Fourier map and refined with the O–H and H⋯H distances constrained to 0.85 (1) and 1.39 (1) Å, respectively, and with *U*_iso_(H) = 1.5*U*_eq_(O). All other H atoms were positioned with idealized geometry and refined isotropically with O—H = 0.83 Å, C—H = 0.95–1.00 Å, and with *U*_iso_(H) = 1.2*U*_eq_(C) or 1.5 *U*_eq_(C-methyl, O). A rotating model was used for the methyl and hy­droxy groups.

## Supplementary Material

Crystal structure: contains datablock(s) cp124135r_0m_a, I. DOI: 10.1107/S2056989018017498/rz5245sup1.cif

Structure factors: contains datablock(s) I. DOI: 10.1107/S2056989018017498/rz5245Isup2.hkl

CCDC reference: 1884284

Additional supporting information:  crystallographic information; 3D view; checkCIF report

## Figures and Tables

**Table 1:** Hydrogen-bond geometry (Å, °) *Cg*1 and *Cg*2 are the centroids of the C29–C34 and C22–C27 rings, respectively.

*D*—H⋯*A*	*D*—H	H⋯*A*	*D*⋯*A*	*D*—H⋯*A*
O1—H1⋯O4^i^	0.82 (4)	2.01 (4)	2.654 (3)	134 (4)
O1*W*—H*WA*⋯O1	0.85 (2)	2.03 (3)	2.838 (3)	159 (2)
O1*W*—H*WB*⋯O8	0.85 (2)	2.27 (3)	3.062 (3)	154 (2)
O3—H3⋯O1*W* ^ii^	0.83 (4)	1.86 (4)	2.680 (3)	174 (4)
C19—H19*B*⋯O3	0.98	2.51	3.445 (3)	159
C1—H1*A*⋯*Cg*1^iii^	0.99	2.98	3.910 (3)	157
C34—H34⋯*Cg*2^iv^	0.95	2.85	3.655 (3)	143

Symmetry codes: (i) 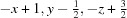; (ii) 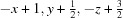; (iii) 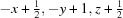; (iv) 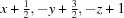.

**Table 2:** Experimental details

Crystal data	
Chemical formula	C_34_H_36_O_8_·H_2_O
*M*_r_	590.64
Crystal system, space group	Orthorhombic, *P*2_1_2_1_2_1_
Temperature (K)	100
*a*, *b*, *c* (Å)	11.8027 (7), 13.2843 (8), 19.0835 (10)
*V* (Å^3^)	2992.1 (3)
*Z*	4
Radiation type	Cu *K*α
μ (mm^−1^)	0.78
Crystal size (mm)	0.35 × 0.24 × 0.10

Data collection	
Diffractometer	Bruker APEXII CCD
Absorption correction	Multi-scan (*SADABS*; Bruker, 2000 ▸)
*T*_min_, *T*_max_	0.772, 0.926
No. of measured, independent and observed [*I* > 2σ(*I*)] reflections	18780, 5425, 4931
*R*_int_	0.061
(sin θ/λ)_max_ (Å^−1^)	0.602

Refinement	
*R*[*F* ^2^ > 2σ(*F* ^2^)], *wR*(*F* ^2^), *S*	0.040, 0.098, 1.03
No. of reflections	5425
No. of parameters	401
No. of restraints	3
H-atom treatment	H atoms treated by a mixture of independent and constrained refinement
Δρ_max_, Δρ_min_ (e Å^−3^)	0.20, −0.23
Absolute structure	Flack *x* determined using 1939 quotients [(*I* ^+^)−(*I* ^−^)]/[(*I* ^+^)+(*I* ^−^)] (Parsons *et al.*, 2013 ▸)
Absolute structure parameter	0.02 (9)

Computer programs: *APEX2* and *SAINT* (Bruker, 2000 ▸), *SHELXT2014* (Sheldrick, 2015*a* ▸), *SHELXL2018* (Sheldrick, 2015*b* ▸) and *SHELXTL* (Sheldrick, 2008 ▸).

## supplementary crystallographic information

### Crystal data

**Table:**

C_34_H_36_O_8_·H_2_O	*D*_x_ = 1.311 Mg m^−^^3^
*M**_r_* = 590.64	Cu *K*α radiation, λ = 1.54178 Å
Orthorhombic, *P*2_1_2_1_2_1_	Cell parameters from 9847 reflections
*a* = 11.8027 (7) Å	θ = 4.1–68.2°
*b* = 13.2843 (8) Å	µ = 0.78 mm^−^^1^
*c* = 19.0835 (10) Å	*T* = 100 K
*V* = 2992.1 (3) Å^3^	Block, colourless
*Z* = 4	0.35 × 0.24 × 0.10 mm
*F*(000) = 1256	

### Data collection

**Table:**

Bruker APEXII CCD diffractometer	4931 reflections with *I* > 2σ(*I*)
φ and ω scans	*R*_int_ = 0.061
Absorption correction: multi-scan (SADABS; Bruker, 2000)	θ_max_ = 68.2°, θ_min_ = 4.1°
*T*_min_ = 0.772, *T*_max_ = 0.926	*h* = −14→14
18780 measured reflections	*k* = −12→16
5425 independent reflections	*l* = −22→22

### Refinement

**Table:**

Refinement on *F*^2^	Hydrogen site location: mixed
Least-squares matrix: full	H atoms treated by a mixture of independent and constrained refinement
*R*[*F*^2^ > 2σ(*F*^2^)] = 0.040	*w* = 1/[σ^2^(*F*_o_^2^) + (0.0537*P*)^2^ + 0.4392*P*] where *P* = (*F*_o_^2^ + 2*F*_c_^2^)/3
*wR*(*F*^2^) = 0.098	(Δ/σ)_max_ < 0.001
*S* = 1.03	Δρ_max_ = 0.20 e Å^−^^3^
5425 reflections	Δρ_min_ = −0.23 e Å^−^^3^
401 parameters	Absolute structure: Flack *x* determined using 1939 quotients [(*I*^+^)-(*I*^-^)]/[(*I*^+^)+(*I*^-^)] (Parsons *et al.*, 2013)
3 restraints	Absolute structure parameter: 0.02 (9)

### Special details

**Table:**

Geometry. All esds (except the esd in the dihedral angle between two l.s. planes) are estimated using the full covariance matrix. The cell esds are taken into account individually in the estimation of esds in distances, angles and torsion angles; correlations between esds in cell parameters are only used when they are defined by crystal symmetry. An approximate (isotropic) treatment of cell esds is used for estimating esds involving l.s. planes.

### Fractional atomic coordinates and isotropic or equivalent isotropic displacement parameters (Å^2^)

**Table:**

	*x*	*y*	*z*	*U*_iso_*/*U*_eq_	
O1	0.50438 (17)	0.47639 (16)	0.71403 (10)	0.0169 (4)	
H1	0.498 (2)	0.423 (3)	0.7356 (16)	0.025*	
O2	0.01638 (17)	0.25183 (15)	0.67636 (10)	0.0208 (4)	
O1W	0.63748 (19)	0.41758 (16)	0.59717 (11)	0.0264 (5)	
HWA	0.614 (3)	0.442 (2)	0.6356 (7)	0.040*	
HWB	0.610 (3)	0.451 (2)	0.5630 (8)	0.040*	
O3	0.3949 (2)	0.72018 (17)	0.88174 (10)	0.0244 (5)	
H3	0.381 (3)	0.780 (3)	0.8896 (9)	0.037*	
O4	0.44345 (18)	0.78239 (15)	0.77797 (10)	0.0222 (5)	
O5	0.31651 (16)	0.68572 (14)	0.68136 (9)	0.0153 (4)	
O6	0.41856 (17)	0.78789 (15)	0.61026 (10)	0.0196 (4)	
O7	0.32786 (16)	0.59542 (15)	0.55359 (9)	0.0164 (4)	
O8	0.50711 (18)	0.57755 (17)	0.51448 (10)	0.0243 (5)	
C1	0.3445 (2)	0.4239 (2)	0.82933 (13)	0.0169 (6)	
H1A	0.275590	0.389055	0.846246	0.020*	
H1B	0.399822	0.371907	0.814829	0.020*	
C2	0.3947 (3)	0.4855 (2)	0.88895 (14)	0.0200 (6)	
H2A	0.413298	0.440659	0.928742	0.024*	
H2B	0.338465	0.535594	0.905294	0.024*	
C3	0.5013 (3)	0.5393 (2)	0.86435 (14)	0.0191 (6)	
H3A	0.559558	0.487970	0.853582	0.023*	
H3B	0.530399	0.580736	0.903561	0.023*	
C4	0.4874 (2)	0.6082 (2)	0.79929 (14)	0.0173 (6)	
C5	0.4224 (2)	0.5473 (2)	0.73970 (14)	0.0143 (6)	
C6	0.3989 (2)	0.6071 (2)	0.67137 (14)	0.0147 (6)	
H6	0.471384	0.637824	0.654684	0.018*	
C7	0.3546 (2)	0.5350 (2)	0.61502 (14)	0.0150 (6)	
H7	0.415279	0.485543	0.602790	0.018*	
C8	0.2478 (3)	0.4785 (2)	0.63531 (13)	0.0155 (6)	
H8	0.186764	0.528923	0.644741	0.019*	
C9	0.2713 (2)	0.4192 (2)	0.70451 (13)	0.0155 (6)	
H9	0.334191	0.370940	0.694319	0.019*	
C10	0.3135 (2)	0.4897 (2)	0.76501 (14)	0.0148 (6)	
C11	0.1683 (2)	0.3559 (2)	0.72822 (14)	0.0179 (6)	
H11A	0.194840	0.298367	0.756862	0.021*	
H11B	0.117878	0.397728	0.757681	0.021*	
C12	0.1045 (3)	0.3179 (2)	0.66718 (15)	0.0178 (6)	
C13	0.1173 (3)	0.3412 (2)	0.59897 (15)	0.0188 (6)	
C14	0.2088 (2)	0.4101 (2)	0.57349 (14)	0.0182 (6)	
H14	0.175084	0.454722	0.536778	0.022*	
C15	0.0311 (3)	0.2860 (2)	0.56192 (15)	0.0232 (7)	
H15	0.017844	0.286084	0.512817	0.028*	
C16	−0.0266 (3)	0.2344 (2)	0.61042 (15)	0.0239 (7)	
H16	−0.088919	0.191433	0.600640	0.029*	
C17	0.4377 (2)	0.7109 (2)	0.81719 (14)	0.0176 (6)	
C18	0.6083 (2)	0.6352 (2)	0.77395 (15)	0.0210 (6)	
H18A	0.649574	0.573260	0.762598	0.031*	
H18B	0.648405	0.671535	0.811094	0.031*	
H18C	0.603385	0.677712	0.732109	0.031*	
C19	0.2189 (2)	0.5629 (2)	0.78820 (14)	0.0165 (6)	
H19A	0.178146	0.587521	0.746845	0.025*	
H19B	0.252518	0.619990	0.813222	0.025*	
H19C	0.165902	0.527710	0.819244	0.025*	
C20	0.3041 (3)	0.3486 (2)	0.53868 (15)	0.0258 (7)	
H20A	0.271281	0.302184	0.504307	0.039*	
H20B	0.344815	0.310097	0.574548	0.039*	
H20C	0.356908	0.394307	0.515049	0.039*	
C21	0.3382 (2)	0.7741 (2)	0.64816 (14)	0.0171 (6)	
C22	0.2512 (2)	0.8514 (2)	0.66403 (14)	0.0173 (6)	
C23	0.1932 (3)	0.8533 (2)	0.72745 (16)	0.0218 (6)	
H23	0.203683	0.800542	0.760439	0.026*	
C24	0.1201 (3)	0.9321 (2)	0.74262 (17)	0.0256 (7)	
H24	0.081958	0.934114	0.786437	0.031*	
C25	0.1025 (3)	1.0083 (2)	0.69359 (18)	0.0275 (7)	
H25	0.052511	1.062358	0.703969	0.033*	
C26	0.1582 (3)	1.0050 (2)	0.62952 (17)	0.0253 (7)	
H26	0.144897	1.056131	0.595677	0.030*	
C27	0.2331 (3)	0.9277 (2)	0.61472 (15)	0.0216 (6)	
H27	0.272173	0.926462	0.571194	0.026*	
C28	0.4154 (2)	0.6172 (2)	0.51063 (14)	0.0175 (6)	
C29	0.3839 (3)	0.6969 (2)	0.45916 (14)	0.0189 (6)	
C30	0.2788 (2)	0.7442 (2)	0.46095 (14)	0.0194 (6)	
H30	0.223119	0.723328	0.493792	0.023*	
C31	0.2556 (3)	0.8222 (2)	0.41454 (16)	0.0256 (7)	
H31	0.184156	0.855115	0.415744	0.031*	
C32	0.3373 (3)	0.8517 (3)	0.36640 (16)	0.0279 (7)	
H32	0.321630	0.905285	0.334856	0.033*	
C33	0.4411 (3)	0.8041 (3)	0.36394 (16)	0.0267 (7)	
H33	0.496239	0.824739	0.330637	0.032*	
C34	0.4651 (3)	0.7262 (2)	0.40992 (15)	0.0221 (6)	
H34	0.536337	0.693019	0.407994	0.027*	

### Atomic displacement parameters (Å^2^)

**Table:**

	*U*^11^	*U*^22^	*U*^33^	*U*^12^	*U*^13^	*U*^23^
O1	0.0187 (11)	0.0154 (10)	0.0167 (9)	0.0038 (9)	0.0027 (8)	0.0020 (7)
O2	0.0228 (11)	0.0219 (11)	0.0177 (10)	−0.0070 (9)	−0.0007 (8)	0.0001 (8)
O1W	0.0306 (13)	0.0244 (12)	0.0243 (10)	0.0037 (10)	0.0089 (9)	0.0046 (9)
O3	0.0342 (13)	0.0197 (11)	0.0194 (10)	0.0030 (10)	0.0037 (9)	−0.0026 (8)
O4	0.0307 (12)	0.0169 (10)	0.0190 (10)	−0.0040 (9)	−0.0041 (9)	0.0005 (8)
O5	0.0152 (10)	0.0142 (10)	0.0166 (9)	0.0018 (8)	0.0008 (8)	0.0007 (7)
O6	0.0203 (11)	0.0204 (11)	0.0180 (9)	−0.0015 (9)	0.0015 (8)	0.0023 (8)
O7	0.0170 (10)	0.0190 (10)	0.0131 (9)	−0.0003 (9)	0.0025 (8)	0.0030 (7)
O8	0.0216 (11)	0.0305 (12)	0.0207 (10)	0.0051 (10)	0.0040 (9)	0.0061 (9)
C1	0.0196 (14)	0.0167 (14)	0.0143 (12)	0.0003 (12)	0.0023 (11)	0.0027 (10)
C2	0.0245 (16)	0.0222 (15)	0.0133 (13)	0.0031 (13)	−0.0010 (11)	0.0018 (11)
C3	0.0223 (16)	0.0172 (15)	0.0178 (13)	0.0023 (13)	−0.0059 (12)	0.0015 (11)
C4	0.0179 (15)	0.0192 (15)	0.0148 (13)	−0.0021 (13)	−0.0019 (11)	−0.0012 (10)
C5	0.0163 (14)	0.0135 (14)	0.0132 (12)	0.0019 (12)	−0.0012 (11)	−0.0007 (10)
C6	0.0141 (13)	0.0141 (13)	0.0158 (12)	0.0001 (12)	0.0025 (11)	0.0025 (10)
C7	0.0177 (15)	0.0152 (14)	0.0120 (12)	0.0036 (12)	0.0005 (11)	0.0020 (10)
C8	0.0168 (14)	0.0152 (14)	0.0144 (12)	0.0016 (12)	0.0017 (11)	−0.0002 (10)
C9	0.0171 (14)	0.0153 (14)	0.0140 (12)	0.0016 (12)	0.0020 (10)	0.0006 (10)
C10	0.0151 (14)	0.0166 (14)	0.0127 (12)	0.0013 (12)	−0.0001 (11)	−0.0008 (10)
C11	0.0200 (15)	0.0180 (14)	0.0156 (13)	−0.0021 (12)	0.0032 (12)	0.0013 (11)
C12	0.0187 (15)	0.0150 (13)	0.0197 (13)	0.0001 (12)	0.0038 (12)	−0.0023 (11)
C13	0.0209 (16)	0.0181 (15)	0.0174 (13)	−0.0008 (13)	−0.0018 (12)	−0.0025 (11)
C14	0.0205 (15)	0.0200 (15)	0.0140 (12)	−0.0032 (13)	0.0002 (11)	0.0004 (11)
C15	0.0260 (16)	0.0253 (17)	0.0183 (14)	−0.0061 (14)	−0.0014 (12)	−0.0023 (12)
C16	0.0235 (16)	0.0272 (17)	0.0210 (15)	−0.0087 (14)	−0.0038 (12)	−0.0047 (12)
C17	0.0156 (14)	0.0205 (15)	0.0169 (13)	−0.0035 (12)	−0.0052 (11)	−0.0022 (11)
C18	0.0172 (15)	0.0243 (16)	0.0214 (14)	−0.0026 (13)	−0.0040 (12)	−0.0002 (12)
C19	0.0163 (14)	0.0184 (14)	0.0147 (12)	0.0009 (12)	0.0022 (11)	0.0001 (10)
C20	0.0312 (18)	0.0277 (17)	0.0184 (14)	−0.0071 (15)	0.0062 (13)	−0.0071 (12)
C21	0.0202 (15)	0.0159 (14)	0.0151 (12)	−0.0016 (12)	−0.0055 (11)	−0.0002 (10)
C22	0.0161 (14)	0.0142 (14)	0.0215 (13)	−0.0022 (12)	−0.0051 (11)	−0.0018 (11)
C23	0.0199 (15)	0.0183 (14)	0.0272 (15)	−0.0027 (13)	−0.0025 (12)	−0.0001 (12)
C24	0.0190 (15)	0.0223 (16)	0.0355 (17)	−0.0038 (14)	0.0013 (13)	−0.0082 (13)
C25	0.0169 (15)	0.0181 (16)	0.0476 (19)	0.0021 (14)	−0.0073 (15)	−0.0073 (14)
C26	0.0229 (16)	0.0172 (16)	0.0359 (17)	0.0014 (14)	−0.0126 (14)	0.0026 (12)
C27	0.0199 (15)	0.0198 (15)	0.0249 (14)	−0.0016 (13)	−0.0072 (12)	−0.0002 (12)
C28	0.0199 (15)	0.0181 (15)	0.0144 (12)	−0.0009 (13)	0.0002 (11)	−0.0020 (10)
C29	0.0257 (16)	0.0180 (15)	0.0129 (12)	−0.0036 (13)	0.0001 (11)	−0.0013 (11)
C30	0.0218 (15)	0.0204 (15)	0.0161 (13)	−0.0030 (13)	0.0020 (11)	−0.0016 (11)
C31	0.0285 (17)	0.0236 (16)	0.0245 (15)	0.0040 (14)	−0.0038 (13)	0.0008 (12)
C32	0.0396 (19)	0.0244 (16)	0.0197 (14)	−0.0020 (16)	−0.0056 (14)	0.0091 (12)
C33	0.0295 (17)	0.0302 (18)	0.0203 (15)	−0.0072 (15)	0.0031 (13)	0.0058 (12)
C34	0.0210 (15)	0.0265 (17)	0.0188 (14)	−0.0016 (14)	0.0032 (11)	0.0025 (12)

### Geometric parameters (Å, º)

**Table:**

O1—C5	1.436 (3)	C11—H11B	0.9900
O1—H1	0.82 (4)	C12—C13	1.347 (4)
O2—C12	1.372 (4)	C13—C15	1.441 (4)
O2—C16	1.376 (3)	C13—C14	1.496 (4)
O1W—HWA	0.8501 (14)	C14—C20	1.541 (4)
O1W—HWB	0.8501 (14)	C14—H14	1.0000
O3—C17	1.337 (3)	C15—C16	1.337 (4)
O3—H3	0.82 (4)	C15—H15	0.9500
O4—C17	1.211 (3)	C16—H16	0.9500
O5—C21	1.358 (3)	C18—H18A	0.9800
O5—C6	1.440 (3)	C18—H18B	0.9800
O6—C21	1.207 (4)	C18—H18C	0.9800
O7—C28	1.350 (3)	C19—H19A	0.9800
O7—C7	1.455 (3)	C19—H19B	0.9800
O8—C28	1.206 (4)	C19—H19C	0.9800
C1—C2	1.522 (4)	C20—H20A	0.9800
C1—C10	1.551 (4)	C20—H20B	0.9800
C1—H1A	0.9900	C20—H20C	0.9800
C1—H1B	0.9900	C21—C22	1.484 (4)
C2—C3	1.522 (4)	C22—C23	1.391 (4)
C2—H2A	0.9900	C22—C27	1.400 (4)
C2—H2B	0.9900	C23—C24	1.387 (4)
C3—C4	1.551 (4)	C23—H23	0.9500
C3—H3A	0.9900	C24—C25	1.394 (5)
C3—H3B	0.9900	C24—H24	0.9500
C4—C17	1.524 (4)	C25—C26	1.389 (5)
C4—C18	1.549 (4)	C25—H25	0.9500
C4—C5	1.593 (4)	C26—C27	1.384 (4)
C5—C6	1.552 (4)	C26—H26	0.9500
C5—C10	1.572 (4)	C27—H27	0.9500
C6—C7	1.532 (4)	C28—C29	1.491 (4)
C6—H6	1.0000	C29—C30	1.391 (4)
C7—C8	1.517 (4)	C29—C34	1.398 (4)
C7—H7	1.0000	C30—C31	1.390 (4)
C8—C14	1.559 (4)	C30—H30	0.9500
C8—C9	1.562 (3)	C31—C32	1.389 (5)
C8—H8	1.0000	C31—H31	0.9500
C9—C11	1.546 (4)	C32—C33	1.379 (5)
C9—C10	1.567 (4)	C32—H32	0.9500
C9—H9	1.0000	C33—C34	1.386 (4)
C10—C19	1.546 (4)	C33—H33	0.9500
C11—C12	1.476 (4)	C34—H34	0.9500
C11—H11A	0.9900		
			
C5—O1—H1	109.5	C15—C13—C14	131.5 (3)
C12—O2—C16	105.7 (2)	C13—C14—C20	110.0 (2)
HWA—O1W—HWB	109.7 (3)	C13—C14—C8	108.9 (2)
C17—O3—H3	109.5	C20—C14—C8	114.8 (2)
C21—O5—C6	116.0 (2)	C13—C14—H14	107.6
C28—O7—C7	116.2 (2)	C20—C14—H14	107.6
C2—C1—C10	112.4 (2)	C8—C14—H14	107.6
C2—C1—H1A	109.1	C16—C15—C13	106.3 (3)
C10—C1—H1A	109.1	C16—C15—H15	126.8
C2—C1—H1B	109.1	C13—C15—H15	126.8
C10—C1—H1B	109.1	C15—C16—O2	111.1 (3)
H1A—C1—H1B	107.9	C15—C16—H16	124.5
C1—C2—C3	110.1 (2)	O2—C16—H16	124.5
C1—C2—H2A	109.6	O4—C17—O3	121.2 (3)
C3—C2—H2A	109.6	O4—C17—C4	122.8 (3)
C1—C2—H2B	109.6	O3—C17—C4	115.7 (2)
C3—C2—H2B	109.6	C4—C18—H18A	109.5
H2A—C2—H2B	108.2	C4—C18—H18B	109.5
C2—C3—C4	115.9 (2)	H18A—C18—H18B	109.5
C2—C3—H3A	108.3	C4—C18—H18C	109.5
C4—C3—H3A	108.3	H18A—C18—H18C	109.5
C2—C3—H3B	108.3	H18B—C18—H18C	109.5
C4—C3—H3B	108.3	C10—C19—H19A	109.5
H3A—C3—H3B	107.4	C10—C19—H19B	109.5
C17—C4—C18	102.6 (2)	H19A—C19—H19B	109.5
C17—C4—C3	112.9 (2)	C10—C19—H19C	109.5
C18—C4—C3	106.8 (2)	H19A—C19—H19C	109.5
C17—C4—C5	115.5 (2)	H19B—C19—H19C	109.5
C18—C4—C5	109.8 (2)	C14—C20—H20A	109.5
C3—C4—C5	108.8 (2)	C14—C20—H20B	109.5
O1—C5—C6	99.8 (2)	H20A—C20—H20B	109.5
O1—C5—C10	109.7 (2)	C14—C20—H20C	109.5
C6—C5—C10	111.2 (2)	H20A—C20—H20C	109.5
O1—C5—C4	104.6 (2)	H20B—C20—H20C	109.5
C6—C5—C4	115.2 (2)	O6—C21—O5	124.0 (3)
C10—C5—C4	114.9 (2)	O6—C21—C22	124.1 (3)
O5—C6—C7	108.4 (2)	O5—C21—C22	111.9 (2)
O5—C6—C5	112.4 (2)	C23—C22—C27	119.8 (3)
C7—C6—C5	109.3 (2)	C23—C22—C21	122.1 (3)
O5—C6—H6	108.9	C27—C22—C21	118.0 (3)
C7—C6—H6	108.9	C24—C23—C22	120.1 (3)
C5—C6—H6	108.9	C24—C23—H23	120.0
O7—C7—C8	107.4 (2)	C22—C23—H23	120.0
O7—C7—C6	107.1 (2)	C23—C24—C25	120.1 (3)
C8—C7—C6	114.4 (2)	C23—C24—H24	120.0
O7—C7—H7	109.3	C25—C24—H24	120.0
C8—C7—H7	109.3	C26—C25—C24	119.8 (3)
C6—C7—H7	109.3	C26—C25—H25	120.1
C7—C8—C14	109.9 (2)	C24—C25—H25	120.1
C7—C8—C9	108.5 (2)	C27—C26—C25	120.4 (3)
C14—C8—C9	113.5 (2)	C27—C26—H26	119.8
C7—C8—H8	108.3	C25—C26—H26	119.8
C14—C8—H8	108.3	C26—C27—C22	119.8 (3)
C9—C8—H8	108.3	C26—C27—H27	120.1
C11—C9—C8	112.5 (2)	C22—C27—H27	120.1
C11—C9—C10	111.0 (2)	O8—C28—O7	123.8 (3)
C8—C9—C10	112.2 (2)	O8—C28—C29	125.0 (3)
C11—C9—H9	106.9	O7—C28—C29	111.2 (2)
C8—C9—H9	106.9	C30—C29—C34	120.1 (3)
C10—C9—H9	106.9	C30—C29—C28	121.8 (3)
C19—C10—C1	107.4 (2)	C34—C29—C28	118.0 (3)
C19—C10—C9	110.9 (2)	C31—C30—C29	119.8 (3)
C1—C10—C9	108.8 (2)	C31—C30—H30	120.1
C19—C10—C5	111.9 (2)	C29—C30—H30	120.1
C1—C10—C5	108.9 (2)	C32—C31—C30	119.7 (3)
C9—C10—C5	108.9 (2)	C32—C31—H31	120.2
C12—C11—C9	110.9 (2)	C30—C31—H31	120.2
C12—C11—H11A	109.5	C33—C32—C31	120.6 (3)
C9—C11—H11A	109.5	C33—C32—H32	119.7
C12—C11—H11B	109.5	C31—C32—H32	119.7
C9—C11—H11B	109.5	C32—C33—C34	120.2 (3)
H11A—C11—H11B	108.1	C32—C33—H33	119.9
C13—C12—O2	110.9 (3)	C34—C33—H33	119.9
C13—C12—C11	128.8 (3)	C33—C34—C29	119.6 (3)
O2—C12—C11	120.3 (2)	C33—C34—H34	120.2
C12—C13—C15	106.1 (3)	C29—C34—H34	120.2
C12—C13—C14	122.4 (3)		
			
C10—C1—C2—C3	−59.2 (3)	C10—C9—C11—C12	160.0 (2)
C1—C2—C3—C4	56.0 (3)	C16—O2—C12—C13	−0.1 (3)
C2—C3—C4—C17	80.5 (3)	C16—O2—C12—C11	178.0 (3)
C2—C3—C4—C18	−167.5 (2)	C9—C11—C12—C13	−8.6 (4)
C2—C3—C4—C5	−49.1 (3)	C9—C11—C12—O2	173.6 (2)
C17—C4—C5—O1	158.7 (2)	O2—C12—C13—C15	0.0 (3)
C18—C4—C5—O1	43.5 (3)	C11—C12—C13—C15	−178.0 (3)
C3—C4—C5—O1	−73.1 (3)	O2—C12—C13—C14	−178.6 (2)
C17—C4—C5—C6	50.3 (3)	C11—C12—C13—C14	3.4 (5)
C18—C4—C5—C6	−65.0 (3)	C12—C13—C14—C20	104.1 (3)
C3—C4—C5—C6	178.5 (2)	C15—C13—C14—C20	−74.1 (4)
C17—C4—C5—C10	−80.9 (3)	C12—C13—C14—C8	−22.5 (4)
C18—C4—C5—C10	163.8 (2)	C15—C13—C14—C8	159.2 (3)
C3—C4—C5—C10	47.3 (3)	C7—C8—C14—C13	169.4 (2)
C21—O5—C6—C7	−99.4 (2)	C9—C8—C14—C13	47.7 (3)
C21—O5—C6—C5	139.7 (2)	C7—C8—C14—C20	45.6 (3)
O1—C5—C6—O5	179.7 (2)	C9—C8—C14—C20	−76.1 (3)
C10—C5—C6—O5	64.0 (3)	C12—C13—C15—C16	0.2 (4)
C4—C5—C6—O5	−69.0 (3)	C14—C13—C15—C16	178.6 (3)
O1—C5—C6—C7	59.3 (3)	C13—C15—C16—O2	−0.3 (4)
C10—C5—C6—C7	−56.4 (3)	C12—O2—C16—C15	0.3 (3)
C4—C5—C6—C7	170.6 (2)	C18—C4—C17—O4	48.5 (3)
C28—O7—C7—C8	−153.2 (2)	C3—C4—C17—O4	163.0 (3)
C28—O7—C7—C6	83.4 (3)	C5—C4—C17—O4	−70.8 (4)
O5—C6—C7—O7	54.0 (3)	C18—C4—C17—O3	−126.3 (2)
C5—C6—C7—O7	176.8 (2)	C3—C4—C17—O3	−11.8 (4)
O5—C6—C7—C8	−64.9 (3)	C5—C4—C17—O3	114.4 (3)
C5—C6—C7—C8	57.9 (3)	C6—O5—C21—O6	2.4 (4)
O7—C7—C8—C14	59.6 (3)	C6—O5—C21—C22	−177.6 (2)
C6—C7—C8—C14	178.4 (2)	O6—C21—C22—C23	−150.1 (3)
O7—C7—C8—C9	−175.8 (2)	O5—C21—C22—C23	30.0 (4)
C6—C7—C8—C9	−57.0 (3)	O6—C21—C22—C27	26.0 (4)
C7—C8—C9—C11	−178.0 (2)	O5—C21—C22—C27	−153.9 (2)
C14—C8—C9—C11	−55.5 (3)	C27—C22—C23—C24	−1.8 (4)
C7—C8—C9—C10	56.0 (3)	C21—C22—C23—C24	174.2 (3)
C14—C8—C9—C10	178.5 (2)	C22—C23—C24—C25	1.5 (4)
C2—C1—C10—C19	−64.1 (3)	C23—C24—C25—C26	0.1 (5)
C2—C1—C10—C9	175.8 (2)	C24—C25—C26—C27	−1.5 (5)
C2—C1—C10—C5	57.2 (3)	C25—C26—C27—C22	1.2 (4)
C11—C9—C10—C19	−59.9 (3)	C23—C22—C27—C26	0.4 (4)
C8—C9—C10—C19	67.0 (3)	C21—C22—C27—C26	−175.7 (3)
C11—C9—C10—C1	58.0 (3)	C7—O7—C28—O8	11.8 (4)
C8—C9—C10—C1	−175.2 (2)	C7—O7—C28—C29	−167.4 (2)
C11—C9—C10—C5	176.6 (2)	O8—C28—C29—C30	−175.4 (3)
C8—C9—C10—C5	−56.6 (3)	O7—C28—C29—C30	3.7 (4)
O1—C5—C10—C19	−175.8 (2)	O8—C28—C29—C34	2.2 (4)
C6—C5—C10—C19	−66.5 (3)	O7—C28—C29—C34	−178.7 (3)
C4—C5—C10—C19	66.6 (3)	C34—C29—C30—C31	−1.3 (4)
O1—C5—C10—C1	65.6 (3)	C28—C29—C30—C31	176.3 (3)
C6—C5—C10—C1	175.0 (2)	C29—C30—C31—C32	0.4 (4)
C4—C5—C10—C1	−51.9 (3)	C30—C31—C32—C33	0.4 (5)
O1—C5—C10—C9	−52.9 (3)	C31—C32—C33—C34	−0.3 (5)
C6—C5—C10—C9	56.5 (3)	C32—C33—C34—C29	−0.5 (5)
C4—C5—C10—C9	−170.4 (2)	C30—C29—C34—C33	1.3 (4)
C8—C9—C11—C12	33.3 (3)	C28—C29—C34—C33	−176.3 (3)

### Hydrogen-bond geometry (Å, º)

Cg1 and Cg2 are the centroids of the C29–C34 and C22–C27 rings, respectively.

**Table:**

*D*—H···*A*	*D*—H	H···*A*	*D*···*A*	*D*—H···*A*
O1—H1···O4^i^	0.82 (4)	2.01 (4)	2.654 (3)	134 (4)
O1*W*—H*WA*···O1	0.85 (2)	2.03 (3)	2.838 (3)	159 (2)
O1*W*—H*WB*···O8	0.85 (2)	2.27 (3)	3.062 (3)	154 (2)
O3—H3···O1*W*^ii^	0.83 (4)	1.86 (4)	2.680 (3)	174 (4)
C19—H19*B*···O3	0.98	2.51	3.445 (3)	159
C1—H1*A*···*Cg*1^iii^	0.99	2.98	3.910 (3)	157
C34—H34···*Cg*2^iv^	0.95	2.85	3.655 (3)	143

Symmetry codes: (i) −*x*+1, *y*−1/2, −*z*+3/2; (ii) −*x*+1, *y*+1/2, −*z*+3/2; (iii) −*x*+1/2, −*y*+1, *z*+1/2; (iv) *x*+1/2, −*y*+3/2, −*z*+1.
